# An extreme value analysis of water levels at the Akosombo dam, Ghana

**DOI:** 10.1016/j.heliyon.2024.e34076

**Published:** 2024-07-08

**Authors:** Saralees Nadarajah, Charles Kwofie

**Affiliations:** a Department of Mathematics, University of Manchester, Manchester M13 9PL, UK; b Department of Mathematics and Statistics, University of Energy and Natural Resources, Ghana

**Keywords:** Cyclic trend, Generalized extreme value distribution, Linear trend

## Abstract

The Akosombo Dam is the largest dam in Ghana and is linked to the world's largest man-made lake by surface area. The top of flood control pool of the dam has been breached a number of times, so it is of interest to know the corresponding probability. The paper fits the generalized extreme value distribution to the extreme water levels – with all three of its parameters (including the shape parameter) accounting for various linear trends, seasonality and cyclic trends with respect to time, *the first time such a model has been fitted*. The fitted model contains in total 50 parameters. It provided an adequate fit, as evaluated by probability plots, quantile plots, and the Kolmogorov-Smirnov test. It is used to provide return level estimates as well as probabilities of the top of flood control pool of the dam being breached.

## 1 Introduction

The Akosombo Dam, the largest dam in Ghana, is situated on the Volta Lake, which holds the title of the world's largest man-made lake by surface area. Constructed under the leadership of Dr. Kwame Nkrumah, Ghana's first president, the primary purpose of the dam was to provide electricity to the country's aluminum industry. However, after some time it became the main source of electricity provider for industries and households. For several years, the dam provided majority of the electricity to neighboring Togo and Benin, although this reliance has significantly reduced presently.

The Akosombo Dam generates 80 percent of Ghana's electricity. Before its construction, less than three percent of the Ghanaian population had access to electricity. Today, an estimated 60 percent of the population has access to electricity.

The Akosombo Dam has significantly boosted fishing and water transportation upstream. Fishing on the dam has become a lucrative business in southern Ghana. Additionally, farming activities have intensified along the 5,500 km shoreline of the dam, with many farmers using its water for irrigation.

The structural height of the Akosombo dam is 375 feet. The top of exclusive flood control pool of the dam is 278 feet. The water levels of the dam can be greater than 278 feet but should not exceed 375 feet. When the water level is greater than 278 feet the excess water can be spilled out by opening the flood control. More about the design of the Akosombo dam can be found in https://vra.com/our_mandate/akosombo_hydro_plant.php.

Because of flooding, it is important that an assessment is made of the water levels in the Akosombo dam. Flooding caused by spillage of the Akosombo dam with its associated effects on neighboring communities are well documented; see, for example, [1] and https://www.ukessays.com/essays/environmental-sciences/case-study-of-the-akosombo-hydroelectric-dam-environmental-sciences-essay.php?vref=1. According to [2], the flooding caused by the dam has displaced many people and significantly impacted the local environment. This includes seismic activity leading to coastal erosion and altered hydrology resulting in microclimatic changes, such as reduced rainfall and higher temperatures.

The water levels at the Akosombo dam have been analyzed by several authors. [3] fitted the generalized Pareto distribution without considering non-stationary features of the data. [4] applied the generalized extreme value distribution, also without accounting for non-stationary features. [5] employed principal components regression and time series analysis to predict the water levels.

The aim of this to paper to estimate the probability of the top of flood control pool of the dam being breached which clearly entails modeling of extreme water levels. We take extreme water levels as the highest water levels observed over a certain period of time. Suppose $Y_{1},\ldots ,Y_{m}$ are observed water levels over *m* days. The extreme water level is $\max\left(Y_{1},\ldots ,Y_{m}\right)=X$ say. Under suitable conditions, a normalized version of the distribution of *X* can be shown to converge to one of the Gumbel, Fréchet, or Weibull distributions, as specified by their cumulative distribution functions [6], [7]

$$\text{Gumbel: }\;\exp\left[-\exp\left(-\frac{x-\mu }{\sigma }\right)\right],$$

$$\text{Fréchet: }\;\exp\left[-{\left(\frac{x-\mu }{\sigma }\right)}^{-\alpha }\right],$$

and

$$\text{Weibull: }\;\exp\left[-{\left(-\frac{x-\mu }{\sigma }\right)}^{\alpha }\right],$$

respectively, as m→∞ for −∞<μ<∞, σ>0 and α>0. [8] demonstrated that the Gumbel, Fréchet, and Weibull distributions can be unified into a single distribution known as the generalized extreme value distribution, which is defined by the cumulative distribution function

(1) $$\exp\left[-{\left(1+\xi \frac{x-\mu }{\sigma }\right)}^{-\frac{1}{\xi }}\right]$$

for $\mu -\frac{\sigma }{\xi }\leq x<\infty$ if ξ>0, −∞<x<∞ if ξ=0 and $-\infty <x\leq \mu -\frac{\sigma }{\xi }$ if ξ<0, where −∞<μ<∞ denotes a location parameter, σ>0 denotes a scale parameter and −∞<ξ<∞ denotes a shape parameter. Note that if ξ>0 then *X* has a heavy tail bounded below by $\mu -\frac{\sigma }{\xi }$. If ξ<0 then *X* has a short tail bounded above by $\mu -\frac{\sigma }{\xi }$.

If *m* is sufficiently large, the distribution of *X*, the extreme water level, can be approximated by (1). This approximation is known as the generalized extreme value model. The properties of this model, including estimation methods, prediction methods, simulation methods, and extensions, have been extensively studied by numerous authors. For detailed information, we refer readers to [9], [10], [11], [12], [13], [14], [15], [16], [17], [18], [19], [20], [21], [22], [23] and the references therein.

In this paper, we fit the generalized extreme value model to the data, allowing all its parameters to vary either linearly or sinusoidally over time. Each parameter of the model including its shape parameter was shown to have at least one significant sinusoidal component. (The generalized extreme value model with constant shape parameter did not give an adequate fit.) Some of the parameters were found to have a significant linear component too. The fitted model was used to infer return level estimates of the water level as well as probability of the top of flood control pool of the dam being breached.

Other studies have utilized the generalized extreme value model to address non-stationary features. For instance, [24] examined extreme temperatures in a mountainous region of Greece, [25] focused on annual maximum precipitation in Oued El Gourzi Watershed, Algeria, and [26] investigated power loss during blackouts in China. However, to our knowledge, there are no papers that have specifically modeled each parameter of the generalized extreme value distribution, particularly its shape parameter, to account for non-stationary features.

The paper is organized as follows: Section 2 describes the data. Section 3 presents the generalized extreme value model, which accommodates non-stationary features. Section 4 provides the results of fitting the model and discusses these findings. Finally, Section 5 offers some conclusions.

## 2 Data

The data consist of daily water level measurements at the Akosombo Dam from 1 January 1965 to 31 December 2013. No data are publicly available beyond this period. The unit of measurement is feet. The data are negatively skewed and heavy tailed.

There are five occurrences of the water level exceeding 278 feet. These occurred at 3712, 4871, 7599, 7600 and 10079 days counting from the 1st of January 1965. They corresponded to the water levels being 284.84, 290.90, 340.30, 340.26 and 290.92, respectively. However, these observations may due to errors in data processing because, for example, the water level on the day immediately preceding 7599 was 240.35. Hence, we shall remove these observations from the data in our extreme value analysis. Table 1 gives summary statistics after removing these five occurrences.

**Table 1:** Some summary statistics of daily water level.

Statistic	Value after removing
	5 largest data
minimum	197.4
first quartile	248.6
median	257.2
mean	256.7
third quartile	266.4
maximum	277.5
skewness	-1.167
kurtosis	6.520
variance	156.814

**Figure 1:**
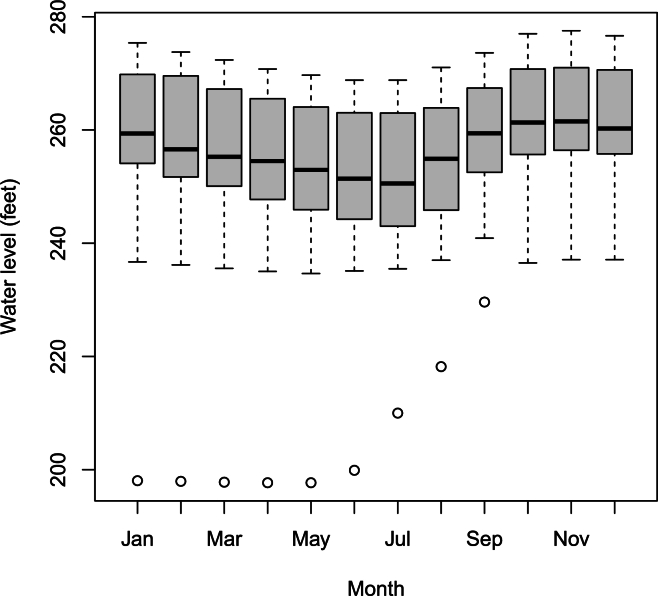
Boxplot of monthly maximum water level versus month.

**Figure 2:**
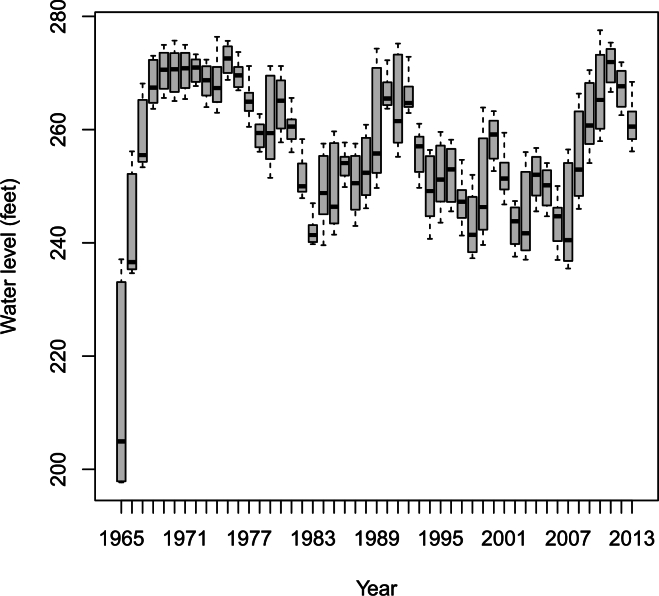
Boxplot of monthly maximum water level versus year.

In the remainder of this paper, we will focus on the monthly maximum water levels. There are 12×49−4=584 maxima recorded over the period from 1 January 1965 to 31 December 2013. Boxplots of these maxima versus months and years are shown in Figure 1, Figure 2. We see non-stationary features: i) the water level is seasonal with minimum occurring in June-July and maximum occurring in December-January; ii) the water level exhibits a cyclic pattern with respect to year. The amplitude of the cycle does not appear constant throughout time. The amplitude appears larger for the initial years.

Section 3 posits that the monthly maximum water levels follow the generalized extreme value model. Estimating this model using the method of maximum likelihood requires the data to be independent, as the likelihood function is defined as the product of probability density functions. We tested for independence using several methods: [27]'s test, [28]'s test, the difference sign test, the rank test, [29]'s test, the turning point test, and [30]'s test. The corresponding *p*-values for these tests were 0.144, 0.159, 0.173, 0.050, 0.112, 0.064, and 0.055, respectively.

## 3 Method

**Figure 3:**
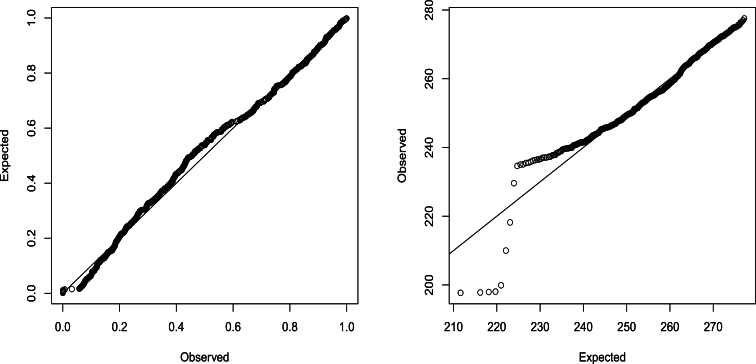
Probability plot (left) and quantile plot (right) for the fit of the generalized extreme value model (1).

Let *X* denote a random variable representing the monthly maximum water level. Fitting the generalized extreme value model to the data on *X* using the method of maximum likelihood (refer to [14] for details) yielded $\overset{ˆ}{\xi }=-0.577$, $\overset{ˆ}{\sigma }=0.014$ and $\overset{ˆ}{\mu }=0.254$ with log⁡L=1783.347. Fig. 3 presents the probability and quantile plots corresponding to this fit. It is evident that the fit of the generalized extreme value model is poor.

To achieve a better fit, we now incorporate the non-stationary features of the data discussed in Section 2. We model the location, scale, and shape parameters as follows:

(2) $$\mu (x)=\mu_{0}+\mu_{1}\cdot \text{month no }(x)+\mu_{2}\cdot \text{year no }(x)+\mu_{3}\sin\left(\pi \frac{\text{month no }(x)}{12}\right)\;\;+\mu_{4}\cos\left(\pi \frac{\text{month no }(x)}{12}\right)\;\;+\sum_{i=1}^{10}\left[\mu_{4i+1}\sin\left(\mu_{4i+2}\cdot \text{year no }(x)\right)+\mu_{4i+3}\cos\left(\mu_{4i+4}\cdot \text{year no }(x)\right)\right],$$

(3) $$\sigma (x)=\exp\{\sigma_{0}+\sigma_{1}\cdot \text{month no }(x)+\sigma_{2}\cdot \text{year no }(x)+\sigma_{3}\sin\left(\pi \frac{\text{month no }(x)}{12}\right)\;\;+\sigma_{4}\cos\left(\pi \frac{\text{month no }(x)}{12}\right)\;\;+\sum_{i=1}^{10}\left[\sigma_{4i+1}\sin\left(\sigma_{4i+2}\cdot \text{year no }(x)\right)+\sigma_{4i+3}\cos\left(\sigma_{4i+4}\cdot \text{year no }(x)\right)\right]\}$$

and

(4) $$\xi (x)=\xi_{0}+\xi_{1}\cdot \text{month no }(x)+\xi_{2}\cdot \text{year no }(x)+\xi_{3}\sin\left(\pi \frac{\text{month no }(x)}{12}\right)\;\;+\xi_{4}\cos\left(\pi \frac{\text{month no }(x)}{12}\right)\;\;+\sum_{i=1}^{10}\left[\xi_{4i+1}\sin\left(\xi_{4i+2}\cdot \text{year no }(x)\right)+\xi_{4i+3}\cos\left(\xi_{4i+4}\cdot \text{year no }(x)\right)\right],$$

respectively, where *x* denotes the data (monthly maximum water level), month no (*x*) taking values 1,2,…,12 (corresponding to the 12 months in a year) denotes the month number corresponding to *x* and year no (*x*) taking values 1,2,…,48 (corresponding to 1965 to 2013) denotes the year number corresponding to *x*. The parameters $\mu_{1}$, $\sigma_{1}$ and $\xi_{1}$ correspond to linear trends with respect to month. The parameters $\mu_{2}$, $\sigma_{2}$ and $\xi_{2}$ correspond to linear trends with respect to year. The parameters $\mu_{3}$, $\mu_{4}$, $\sigma_{3}$, $\sigma_{4}$, $\xi_{3}$ and $\xi_{4}$ correspond to seasonality with respect to month. The remaining parameters are associated with cyclic trends over the years. We have constrained the number of these cyclic trends to 10, resulting in a total of 135 parameters.

The model described by (2), (3) and (4) was fitted using the method of maximum likelihood by maximizing

$$L\left(\mu ,\sigma ,\xi \right)=\prod_{i=1}^{584}\left\{\frac{1}{\sigma \left(x_{i}\right)}{\left(1+\xi \left(x_{i}\right)\frac{x_{i}-\mu \left(x_{i}\right)}{\sigma \left(x_{i}\right)}\right)}^{-1-\frac{1}{\xi \left(x_{i}\right)}}\exp\left[-{\left(1+\xi \left(x_{i}\right)\frac{x_{i}-\mu \left(x_{i}\right)}{\sigma \left(x_{i}\right)}\right)}^{-\frac{1}{\xi \left(x_{i}\right)}}\right]\right\},$$

where $\left\{x_{i},i=1,2,\ldots ,584\right\}$ are the data, $\mu \left(x_{i}\right)$ is given by (2), $\sigma \left(x_{i}\right)$ is given by (3), $\xi \left(x_{i}\right)$ is given by (4), $\mu =\left(\mu_{0},\ldots ,\mu_{44}\right)$, $\sigma =\left(\sigma_{0},\ldots ,\sigma_{44}\right)$ and $\xi =\left(\xi_{0},\ldots ,\xi_{44}\right)$. The maximization was performed by using the optim function in the R software [31]. Let $\overset{ˆ}{\mu }=\left(\overset{ˆ}{\mu_{0}},\ldots ,\overset{ˆ}{\mu_{44}}\right)$, $\overset{ˆ}{\sigma }=\left(\overset{ˆ}{\sigma_{0}},\ldots ,\overset{ˆ}{\sigma_{44}}\right)$ and $\overset{ˆ}{\xi }=\left(\overset{ˆ}{\xi_{0}},\ldots ,\overset{ˆ}{\xi_{44}}\right)$ denote that maximum likelihood estimates of $\overset{ˆ}{\mu }$, $\overset{ˆ}{\sigma }$ and $\overset{ˆ}{\xi }$, respectively.

Standard errors / confidence intervals associated with parameters were obtained by bootstrapping as described in [32].

## 4 Results and discussion

We used the method in Section 3 to model the data on monthly maximum water level. We started with fitting the generalized extreme value model having just three parameters $\left(\mu_{0},\sigma_{0},\xi_{0}\right)$ and then added one parameter at a time to fit the models having $\left(\mu_{0},\mu_{1},\sigma_{0},\xi_{0}\right)$, $\left(\mu_{0},\mu_{1},\sigma_{0},\sigma_{1},\xi_{0}\right)$, $\left(\mu_{0},\mu_{1},\sigma_{0},\sigma_{1},\xi_{0},\xi_{1}\right)$, and so on until no more parameters can be added. We also started with fitting the full model (having 135 parameters) and subtracted one parameter at a time to fit the models having $(\mu_{0},\ldots ,\mu_{43}$, $\sigma_{0},\ldots ,\sigma_{44}$, $\xi_{0},\ldots ,\xi_{44})$, $(\mu_{0},\ldots ,\mu_{43}$, $\sigma_{0},\ldots ,\sigma_{43}$, $\xi_{0},\ldots ,\xi_{44})$, $(\mu_{0},\ldots ,\mu_{43}$, $\sigma_{0},\ldots ,\sigma_{43}$, $\xi_{0},\ldots ,\xi_{43})$, and so on until no more parameters can be deleted. Both approaches resulted in the same model. The significance of parameters to be added or removed was determined using the likelihood ratio test by comparing likelihood values, as described by [33]. Additionally, we used the Akaike information criterion [34] and the Bayesian information criterion [35] to assess the significance of the parameters.

Table 2 presents the parameter estimates and standard errors of the final model. The maximized loglikelihood was log⁡L=2093.913. The standard errors were obtained through bootstrapping. It is observed that all standard errors are smaller in magnitude than the corresponding parameter estimates.

**Table 2:** Parameter estimates and standard errors (obtained by simulation) of the final model.

Parameter	Estimate (se)	Parameter	Estimate (se)	Parameter	Estimate (se)
*μ*_0_	446.333 (32.510)	*σ*_0_	2.509 (0.041)	*ξ*_0_	-0.977 (0.026)
*μ*_1_	-11.934 (0.780)	*σ*_1_	-0.012 (0.001)	*ξ*_1_	0.004 (0.000)
*μ*_2_	-0.579 (0.037)	*σ*_2_	-0.291 (0.027)	*ξ*_2_	0.012 (0.001)
*μ*_3_	-22.622 (1.649)	*σ*_3_	-0.203 (0.002)	*ξ*_3_	-0.137 (0.008)
*μ*_4_	0.020 (0.002)	*σ*_4_	-0.081 (0.005)	*ξ*_4_	-0.053 (0.003)
*μ*_5_	-123.611 (2.185)	*σ*_5_	0.338 (0.020)	*ξ*_5_	0.000 (0.000)
*μ*_6_	-0.005 (0.000)	*σ*_6_	0.299 (0.002)	*ξ*_6_	0.000 (0.000)
*μ*_7_	-59.830 (3.399)	*σ*_7_	0.482 (0.008)	*ξ*_7_	0.003 (0.000)
*μ*_8_	-0.006 (0.000)	*σ*_8_	0.519 (0.050)	*ξ*_8_	0.000 (0.000)
*μ*_9_	-22.108 (2.010)	*σ*_9_	0.492 (0.039)	*ξ*_9_	0.000 (0.000)
*μ*_10_	0.032 (0.001)	*σ*_10_	-0.126 (0.012)	*ξ*_10_	0.000 (0.000)
*μ*_11_	-31.641 (1.454)	*σ*_11_	-0.034 (0.001)	*ξ*_11_	0.000 (0.000)
*μ*_12_	0.033 (0.002)	*σ*_12_	0.080 (0.003)	*ξ*_12_	0.000 (0.000)
*μ*_13_	-25.550 (0.534)	*σ*_13_	-0.055 (0.001)		
*μ*_14_	0.001 (0.000)	*σ*_14_	0.190 (0.007)		
*μ*_15_	-5.113 (0.004)	*σ*_15_	-0.391 (0.007)		
*μ*_16_	0.642 (0.001)	*σ*_16_	0.155 (0.004)		
*μ*_17_	5.217 (0.353)	*σ*_17_	-0.270 (0.015)		
*μ*_18_	0.237 (0.001)				

The identifiability of the final model was tested using the Matlab package Data2Dynamics due to [36]. We also tested for collinearity between month number and year number using the R package rfUtilities [37].

The location parameter exhibits seasonality with respect to month and four cyclic trends with respect to year. The amplitude and phase corresponding to the seasonality term are 11.948 and -0.048, respectively. The scale parameter exhibits a negative trend with respect to year and four cyclic trends with respect to year. The shape parameter exhibits a positive trend with respect to month, a positive trend with respect to year, seasonality with respect to month and two cyclic trends with respect to year. The amplitude and phase corresponding to the seasonality term are 0.147 and 0.370, respectively.

**Figure 4:**
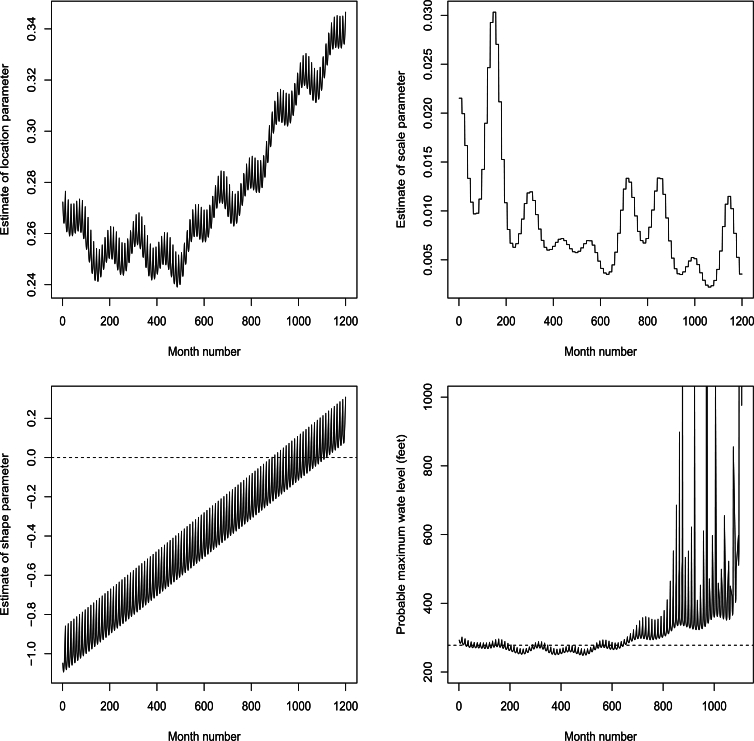
Plots of $\overset{ˆ}{\mu }$ (top left), $\overset{ˆ}{\sigma }$ (top right), $\overset{ˆ}{\xi }$ (bottom left) and $\overset{ˆ}{\mu }-\frac{\overset{ˆ}{\sigma }}{\overset{ˆ}{\xi }}$ (bottom right) versus month number counted from January 1965. $\overset{ˆ}{\mu }-\frac{\overset{ˆ}{\sigma }}{\overset{ˆ}{\xi }}$ is plotted only if $\overset{ˆ}{\xi }<0$. The dashed line corresponds to $\overset{ˆ}{\xi }$ taking the value zero or $\overset{ˆ}{\mu }-\frac{\overset{ˆ}{\sigma }}{\overset{ˆ}{\xi }}$ being equal to 278 feet.

**Figure 5:**
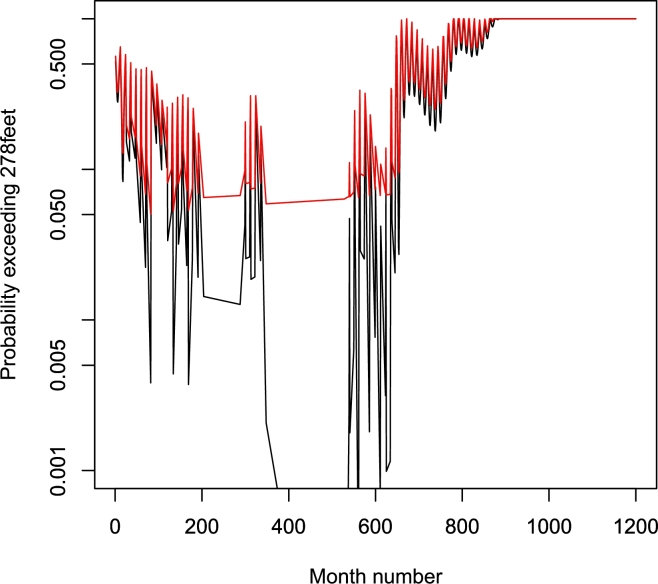
The probability of the top of flood control pool of the dam being breached with simulated 95 percent confidence interval. The lower limit of the confidence interval is zero. The upper limit is in red. The estimated probabilities are in black. Both axes are in log scale.

Plots of $\overset{ˆ}{\mu }$, $\overset{ˆ}{\sigma }$, $\overset{ˆ}{\xi }$ and $\overset{ˆ}{\mu }-\frac{\overset{ˆ}{\sigma }}{\overset{ˆ}{\xi }}$ are shown in Fig. 4. We see that $\overset{ˆ}{\mu }$ generally increases, $\overset{ˆ}{\sigma }$ generally decreases and $\overset{ˆ}{\xi }$ generally increases. $\overset{ˆ}{\xi }<0$ for months ranging from 1 to 1109. Over these months, the monthly maximum water level will have the probable upper bound, $\overset{ˆ}{\mu }-\frac{\overset{ˆ}{\sigma }}{\overset{ˆ}{\xi }}$, also plotted in Fig. 4. We note that the upper bound is less than 278 feet for months in certain intervals between 27 months and 644 months. Hence, the top of flood control pool of the dam will not be breached over these periods. The probability of the top being breached over other periods is shown in Fig. 5. The probability is around 0.05 most of the time. But after 889 months the probability reaches 1.

**Figure 6:**
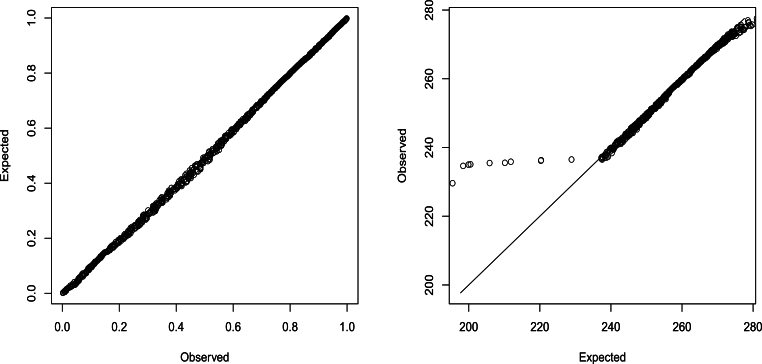
Probability plot (left) and quantile plot (right) of the fitted model.

The probability and quantile plots of the fitted model are shown in Fig. 6, the fits shown by these plots appear much better compared to Fig. 3. The probability plot is the plot of

$$\exp\left[-{\left(1+\overset{ˆ}{\xi \left(x_{\left(i\right)}\right)}\frac{x_{\left(i\right)}-\overset{ˆ}{\mu \left(x_{\left(i\right)}\right)}}{\overset{ˆ}{\sigma \left(x_{\left(i\right)}\right)}}\right)}^{-\frac{1}{\overset{ˆ}{\xi \left(x_{\left(i\right)}\right)}}}\right]$$

versus $\frac{i}{n+1}$ for i=1,2,…,n, where $x_{\left(1\right)}\leq x_{\left(2\right)}\leq \cdots \leq x_{\left(n\right)}$ are data arranged in increasing order and $\overset{ˆ}{\mu \left(x_{\left(i\right)}\right)}$, $\overset{ˆ}{\sigma \left(x_{\left(i\right)}\right)}$ and $\overset{ˆ}{\xi \left(x_{\left(i\right)}\right)}$ are given by (2), (3) and (4), respectively. The quantile plot is the plot of

$$\overset{ˆ}{\mu \left(x_{\left(i\right)}\right)}+\frac{\overset{ˆ}{\sigma \left(x_{\left(i\right)}\right)}}{\overset{ˆ}{\xi \left(x_{\left(i\right)}\right)}}\left\{{\left[-\log\left(\frac{i}{n+1}\right)\right]}^{-\overset{ˆ}{\xi \left(x_{\left(i\right)}\right)}}-1\right\}$$

versus $x_{\left(i\right)}$ for i=1,2,…,n. The probability plot shows that the fitted model is adequate. The quantile plot shows adequacy except in the lower tail. The poor fit in the lower tail may be due to errors in measurements in the early period. The *p*-value of the Kolmogorov-Smirnov test [38], [39] was 0.061.

**Figure 7:**
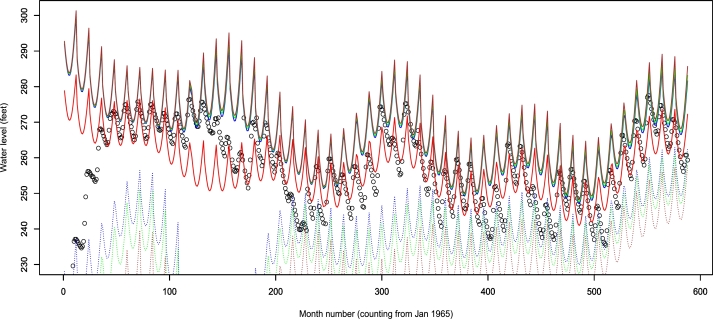
Plot of the data with 50 percent (red), 95 percent (solid blue), 97.5 percent (solid green), 99 percent (solid brown), 5 percent (broken blue), 2.5 percent (broken green) and 1 percent (broken brown) quantiles.

A further diagnostic of the fitted model over the observed data period is shown in Fig. 7. We see that the estimated quantiles closely follow the pattern in the data.

**Figure 8:**
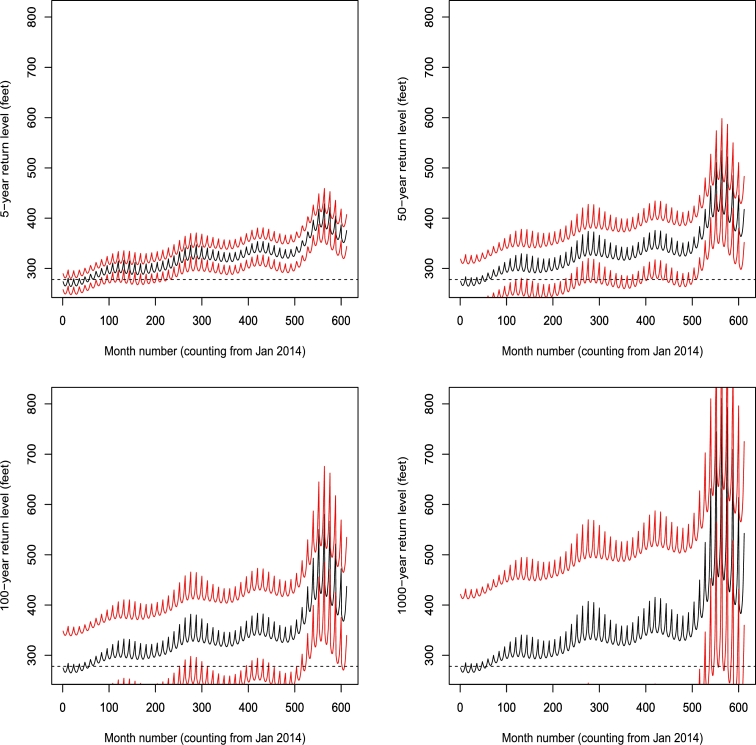
5 (top left), 50 (top right), 100 (bottom left) and 1000 (bottom right) year return levels with simulated 95 percent confidence intervals. The return level estimates are in black. The confidence intervals are in red. The dashed lines correspond to the return levels being equal to 278 feet.

Fig. 8 shows the return levels corresponding to 5, 50, 100 and 1000 years. The return levels are bounded above by the probable upper bound up until about 50 months. Thereafter, we see increasing gaps between the return levels. The probability of breach of the top of flood control pool of the dam decreases up until about 200 months counting from January 2014. Thereafter, the probability increases with time. More water can be released from the dam to minimize the probability of breach. The water can be diverted to areas experiencing droughts in Ghana [40], [41], [43].

Finally, robustness checks were conducted to validate our best-fitting model (2)-(4). This involved splitting the data into two halves and removing the first few observations (see the quantile plot in Fig. 6). Similar checks are discussed in [42] and [43]. We fitted the three-parameter generalized extreme value model to each half of the data and to the reduced data set, incrementally adding one parameter at a time until no further parameters could be added. Additionally, we fitted the full model, comprising 135 parameters, to each half and to the reduced data, systematically subtracting one parameter at a time until no further parameters could be removed.

The first half of the data corresponds to the period from 1 January 1965 to 31 December 1989, while the second half covers 1 January 1990 to 31 December 2013. The best-fitting model for each half and for the reduced data was of the form (2)-(4); that is, the location parameter contained seasonality with respect to month and four cyclic trends, the scale parameter contained a trend with respect to year and four cyclic trends, and the shape parameter contained a trend with respect to month, a trend with respect to year, seasonality with respect to month and two cyclic trends.

## 5 Conclusions

We have conducted an extreme value analysis of water levels at the Akosombo Dam, the largest dam in Ghana. Our analysis has taken account of trends with respect to month, trends with respect to year, seasonality with respect to month and cyclic trends with respect to year. Previous research on this data has not accounted for these features.

The model with 50 parameters was found to provide a satisfactory fit, as demonstrated through probability plots, quantile plots, and the Kolmogorov-Smirnov test. Using the fitted model, we were able to deduce return level estimates as well as probabilities of the top of flood control pool of the dam being breached. The probabilities appear minimal up until 889 months counting from the 1st of January 1965.

The dam is managed by the Volta River Authority, a state-owned enterprise in Ghana responsible for the development, generation, and transmission of electrical power. The VRA oversees the day-to-day operations of the dam. Efficient operation and regular maintenance of the dam's infrastructure are critical for its performance and longevity. This should include monitoring water levels, turbine efficiency, and addressing any issues that may arise. One of the primary purposes of the dam is to generate electricity. The management should focus on optimizing the generation capacity to meet the energy needs of Ghana and the surrounding region. The dam regulates the flow of the Volta River, impacting downstream ecosystems and water availability. Balancing the water release should be essential for both electricity generation and environmental considerations. The management of the dam should include efforts to mitigate environmental impacts such as managing the water levels to minimize downstream flooding and addressing issues related to the displacement of communities during the dam's construction. Given the social and environmental impact of large dams, maintaining positive relations with local communities is crucial. This should involve addressing concerns, providing compensation when necessary, and engaging in sustainable development initiatives. Over time, the dam may undergo upgrades and modernization to enhance efficiency, safety, and environmental performance. Management decisions regarding such upgrades should be made based on technical, economic, and environmental considerations.

The following actions could be taken for disaster preparedness: identify potential hazards in the area of the dam; assess vulnerabilities and determine the potential impact on the community; raise awareness about potential risks and the importance of preparedness; conduct training sessions for community members on evacuation procedures and first aid; develop and communicate emergency plans for different types of disasters; establish evacuation routes and assembly points; ensure there are reliable communication systems in place to disseminate information quickly; establish a system for alerting residents about imminent threats; stockpile essential supplies such as food, water, first aid kits, and blankets; encourage residents to have personal emergency kits; invest in infrastructure that can withstand disasters; regularly inspect and maintain critical infrastructure; foster a sense of community and encourage neighbors to support each other during emergencies; establish volunteer groups for emergency response; implement early warning systems for specific hazards; ensure that residents are familiar with these systems and know how to respond; train and equip local emergency services for rapid response; coordinate with regional and national emergency services for support; regularly review and update emergency plans based on lessons learned and changes in the community.

The infrastructure planning for the dam should involve: maintaining and upgrading the dam's facilities to ensure efficient and reliable power generation; periodic assessments determining the capacity of the dam and the potential for expansion or upgrades to meet increasing energy demands; managing the reservoir to balance its various uses including fishing, transportation, and irrigation; development and maintenance of a robust transmission network to distribute power to urban and rural areas; measures to mitigate environmental impacts such as fish migration pathways, erosion control, and addressing the displacement of communities; development of tourism facilities, recreational areas, and supporting services; initiatives for community development, including education, healthcare, and other essential services; scheduling and implementing maintenance activities to ensure the continued safe operation of the dam; early warning systems and evacuation procedures.

Future work is to perform similar analysis for other important dams in Africa, including the grand Ethiopian renaissance dam, the Katse Arch dam located in the Kingdom of Lesotho, the Aswan High dam in Egypt, the Cahora Bassa dam in Mozambique and the Gibe III dam in Ethiopia. Another work is to have water extraction as a covariate in the analysis. But data on water extraction do not appear available for any of the dams in Africa.

## Ethics approval

Not applicable.

## Funding

No funds received for this research.

## Code availability

The code used can be obtained from the corresponding author.

## CRediT authorship contribution statement

**Saralees Nadarajah:** Investigation. **Charles Kwofie:** Investigation.

## Declaration of Competing Interest

The authors declare that they have no known competing financial interests or personal relationships that could have appeared to influence the work reported in this paper.

## Data Availability

Data will be made available on request from the corresponding author.

## References

[1] Miescher S.F. (2021). Ghana's Akosombo dam, volta lake fisheries and climate change. Daedalus.

[2] Gyau-Boakye P. (2001). Environmental impacts of the Akosombo dam and effects of climate change on the lake levels. Environ. Dev. Sustain..

[3] Minkah R. (2016). An application of extreme value theory to the management of a hydroelectric dam. SpringerPlus.

[4] Ocran E., Doku-Amponsah K., Nortey E.N.N. (2017). Estimating exceedance probability of extreme water levels of the Akosombo dam. CBAS J. Sci. Dev..

[5] Ofori Asare I.O., Frempong D.A., Larbi P. (2018). Use of principal components regression and time-series analysis to predict the water level of the Akosombo dam level. Int. J. Stat. Appl..

[6] Fisher R.A., Tippett L.H.C. (1928). Limiting forms of the frequency distribution of the largest and smallest member of a sample. Proc. Camb. Philos. Soc..

[7] Gnedenko B.V. (1943). Sur la distribution limite du terme maximum d'une serie aleatoire. Ann. Math..

[8] Jenkinson A.F. (1955). The frequency distribution of the annual maximum (or minimum) values of meteorological elements. Q. J. R. Meteorol. Soc..

[9] Leadbetter M.R., Lindgren G., Rootzén H. (1983).

[10] Galambos J. (1987).

[11] Resnick S.I. (1987).

[12] Embrechts P., Klüppelberg C., Mikosch T. (1997).

[13] Kotz S., Nadarajah S. (2000).

[14] Coles S.G. (2001).

[15] Beirlant J., Goegebeur Y., Teugels J., Segers J. (2004).

[16] Gumbel E.J. (2004).

[17] Castillo E., Hadi A.S., Balakrishnan N., Sarabia J.M. (2005).

[18] Malevergne Y., Sornette D. (2006).

[19] de Haan L., Ferreira A. (2007).

[20] Reiss R.-D., Thomas M. (2007).

[21] Falk M., Hüsler J., Reiss R.-D. (2011).

[22] Novak S.Y. (2012).

[23] Ahsanullah M. (2016).

[24] Caroni C., Panagoulia D. (2016). Non-stationary modeling of extreme temperatures in a mountainous area of Greece. REVSTAT Stat. J..

[25] Bella N., Dridi H., Kalla M. (2020). Statistical modeling of annual maximum precipitation in Oued El Gourzi Watershed, Algeria. Appl. Water Sci..

[26] Chen H.L., Zhao T.T.G. (2020). Modeling power loss during blackouts in China using non-stationary generalized extreme value distribution. Energy.

[27] Bartels R. (1982). The rank version of von Neumann's ratio test for randomness. J. Am. Stat. Assoc..

[28] Cox D.R., Stuart A. (1955). Some quick sign test for trend in location and dispersion. Biometrika.

[29] Wald A., Wolfowitz J. (1940). On a test whether two samples are from the same population. Ann. Math. Stat..

[30] Box G.E.P., Pierce D.A. (1970). Distribution of residual correlations in autoregressive-integrated moving average time series models. J. Am. Stat. Assoc..

[31] R Development Core Team (2023).

[32] Babu G.J., Rao C.R. (2004). Goodness-of-fit tests when parameters are estimated. Sankhya.

[33] Cox D.R., Hinkley D.V. (1974).

[34] Akaike H. (1974). A new look at the statistical model identification. IEEE Trans. Autom. Control.

[35] Schwarz G.E. (1978). Estimating the dimension of a model. Ann. Stat..

[36] Kreutz C. (2018). An easy and efficient approach for testing identifiability. Bioinformatics.

[37] Evans J.S., Murphy M.A. (2019).

[38] Kolmogorov A. (1933). Sulla determinazione empirica di una legge di distribuzione. G. Ist. Ital. Attuari.

[39] Smirnov N. (1948). Table for estimating the goodness of fit of empirical distributions. Ann. Math. Stat..

[40] Incoom A.B.M., Adjei K.A., Odai S.N. (2020). Rainfall variabilities and droughts in the Savannah zone of Ghana from 1960-2015. Sci. Afr..

[41] Addi M., Asare K., Fosuhene S.K., Ansah-Narh T., Aidoo K., Botchway C.G. (2021). Impact of large-scale climate indices on meteorological drought of coastal Ghana. Adv. Meteorol..

[42] Chan S., Chu J., Zhang Y., Nadarajah S. (2021). Count regression models for Covid-19. Phys. A, Stat. Mech. Appl..

[43] Nadarajah S., Afuecheta E., Chan S. (2021). Dependence between bitcoin and African currencies. Qual. Quant..

